# Rape Stereotype Acceptance in the General Population of England and Wales

**DOI:** 10.1177/08862605221076162

**Published:** 2022-02-27

**Authors:** Megan Hermolle, Samantha J. Andrews, Ching-Yu S. Huang

**Affiliations:** 1 School of Psychology, 4212 Keele University, Cambridge, UK

**Keywords:** adult victims, anything related to sexual assault, revictimization, sexual assault

## Abstract

The #MeToo movement has facilitated a growing awareness in the UK of rape stereotypes but there has been little research on how accurately rape is perceived in this region, especially regarding demographics such as ethnicity and age. This study recruited 1000 participants, representative of the UK population, to complete an online survey prompting beliefs about rape perpetrators, rape victims, rape allegations, male rape, and motives for and consequences of rape. After carrying out frequency analyses on agree-incorrect and disagree-incorrect statements, we found that, generally, accuracy was high, although there were higher levels of stereotype acceptance for perpetrator related stereotypes. Further analysis found that in terms of demographic differences, Black and Asian participants and men were significantly more likely to accept stereotypes than other demographic groups. Implications for policy and practice are discussed, including potential for jury education, and educational media campaigns aimed at the demographics most likely to accept stereotypes.

## Introduction

The extent and nature of rape stereotype acceptance amongst the general public in England and Wales is a topic that needs updating. Current issues of rape attrition, potentially arising from underlying perceptions or stereotyping, drive the need for new, comprehensive research. Rape has the highest cost to society, with a devastating individual impact and a high social and economic impact (Burgess & Carretta, 2017; Heeks et al., 2018), yet one of the lowest prosecution rates. Only 1.4% of rapes in year 2019 to 2020 resulted in a charge or summons (Home Office, 2020), with even fewer resulting in conviction. Due to possible impacts of rape stereotyping by the general public, who represent juries, and also legal professionals, it is crucial to discover the extent of rape stereotyping at present in the UK.

### The Cost of Rape and Rape Stereotyping

Burt (1980) first defined rape stereotypes, or rape myths, as ‘prejudicial, stereotyped or false beliefs about rape, rape victims and rapists’ (Burt, 1980, p. 217). This study was the first in social psychology to define rape stereotypes with the 19 item Rape Myth Acceptance Scale. It also found strong connections between different variables, including attitudes towards gender roles, personality traits, personal experiences and individual background and people’s acceptance of rape myths. The topic has since been widely researched with new measures created. For example, Briere et al. (1985) conducted an empirical study to assess the complexity of rape stereotypes, creating nine new scales, many of which were significantly associated with stereotype acceptance, while Payne et al. (1999) created and studied the 45-item Illinois Rape Myth Acceptance Scale (IRMA) across several studies.

The UK legal definition of rape is intentionally penetrating the vagina, anus, or mouth of another person with a penis. The victim does not consent to the penetration and the offender does not reasonably believe that the victim consents (Sexual Offences Act, 2003).

This definition of rape is restrictive, as it does not include the rape of men by women. The impact of rape is far-reaching and devastating. Victims may experience physical effects such as sexually transmitted diseases or pregnancy, and psychological consequences such as Post Traumatic Stress Disorder (PTSD), depression, anger and feelings of vulnerability and high levels of self-blame (Burgess & Carretta, 2017, p. 3–9). Although the authors were writing from an American perspective, they pointed out that the way culture defines gender roles impacts the perception of rape and cost to the victim. Similarly to the US, the United Kingdom operates from a history of patriarchy and consequently normalisation of rape, suggesting this is a valuable perspective.

The physical and emotional costs to the individual also contribute to a high economic and social impact. Heeks et al. (2018) carried out a thorough report on the economic and social costs of crime, first estimating the total number of crimes using Home Office and Crime Survey of England and Wales statistics, and then estimating the costs of crime using several criteria. They found that of all non-fatal crimes, rape had the highest estimated cost, at £39,360 per offence. These costs included physical and emotional harms, time taken off work, and preventative measures. The estimated number of crimes was 121,746, leading to a total cost of £4.8 billion for the year. Consequently, up-to-date research on rape and rape stereotyping is needed to explore how these costs to individual and society can be reduced.

Furthermore, this crime has the highest cost to society, yet one of the lowest prosecution rates. The Crime Survey of England and Wales (Office for National Statistics, 2020) found that .05% of men and 7.1% of women aged between 16 and 59 were victims of rape or attempted rape.

Rape is a highly underreported crime, so these estimates are likely to be less than the true figures: approximately 83% of people who had experienced rape had never reported to the police (Office for National Statistics, 2018). The widening gap between rapes, their reports, and prosecutions is concerning – the Crime Outcomes in England and Wales Report revealed that in 2019-20, only 1.4% of rapes resulted in a charge or summons, and 41% of cases resulted in the victim dropping out of the case, leading to high attrition rates (Home Office, 2020). Contributing to this concern is that rape stereotypes contribute to poor investigation and outcomes for rape complainants – Hohl and Stanko (2015) carried out a large-scale representative study sampling rape complaints made to the London Metropolitan Police Service. Discussing the range of factors associated with attrition in their literature review, they pointed out that all factors are bound up in rape stereotypes, and their findings supported this, with significant evidence suggesting that real rapist, victim resistance and ‘respectable woman’ stereotypes are considerable factors in attrition.

Another contributing factor to attrition is likely the digital processing notices which pressure victims to submit personal and often irrelevant information to the police. Many refuse, and consequently, their case is dropped (HM Crown Prosecution Service Inspectorate, 2019), perhaps due to legal officials’ victim blaming and rape stereotyping. Additionally, the End Violence Against Women Coalition (EVAW) recently investigated the Crown Prosecution Service’s (CPS) failure to prosecute rape and found that there was a growing culture within the CPS of risk-avoidance, suggesting that due to a change in approach, the CPS has only been pursuing ‘easy’ cases. This could also be related to acceptance of stereotypes within the CPS, and had a trickle-down effect on the police, causing them to take a similar approach (End Violence Against Women Coalition, 2019).

### Social Representations Theory

It is clear that there is a heavy societal, as well as individual, price for rape, likely perpetuated by stereotypes about ‘real’ rape victims or ‘real’ perpetrators. To aid in our understanding of these complex dynamics, the present research must be situated within an appropriate social psychological framework. Social Representations Theory (Moscovici, 2000) focuses on the social nature of communication, and how social representations influence society through the individual. Therefore, this is a useful lens through which to study the way in which rape stereotypes are not only generated but also perpetuated by society through the individual.

Stereotypes are created when particular images or stories are repeated. When repeated and amplified socially, the concept becomes a generally accepted belief about members of a social category or group (Taylor & Stern, 1997). According to Höijer (2011), this repetition and amplification of concepts and beliefs about groups begins in childhood and are transmitted through traditional institutions, including family, religion, law, and media.

Therefore, within structures such as patriarchy, which influences many cultures, stereotypes about gender and rape are passed down generation to generation. Rape stereotypes have existed for centuries. For example, Lord Justice Matthew Hale in the late 18^th^ century asserted that rape is, ‘an accusation easily to be made and hard to be proved and harder to be defended by the party accused, tho never so innocent’ (Hale, 1778, p.635). Statements such as this have been repeated, used in legal settings, and become reified as rape stereotypes through social representations.

Social Representations Theory originates from Durkheim’s concept of collective representations, a theory of how social reality is navigated. Moscovici (2000) felt this concept was too static for how dynamic and changeable the social conditions of contemporary society are. He emphasised the way in which representations arise through interaction and communication between individuals and groups, reflecting cultural and historical contexts.

This is one likely reason, since the rise of accessible online and print news media, for the media being a substantial agent for perpetuating social representations and stereotypes of real rape and real rapists. For example, O’Hara (2012) carried out a lexical analysis of 124 news articles about three sensationalised rape cases, finding that the perpetrator was often ‘othered’, and described as a ‘beast’ or ‘freak’. This distances the perpetrator from society, yet approximately 90% of rape perpetrators are known to the victim (ONS, 2013).

Additionally, ‘real victim’ stereotypes are often perpetuated by the media through representations of young white virginal women, or drunken ‘slut’ who ‘wanted it’. Benedict (1993) suggested that the latter is a classic victim blaming stereotype, while the former is reductive, exclusionary, and dishonest. This influences court proceedings and jury decision-making already affected by long-established patriarchal social representations of rape: while social representations are ingrained in jurors, rape stereotyping is also routinely used by the defence to undermine the victim or exonerate the perpetrator. Temkin et al. (2018) carried out a court observation study and found a wide range of rape stereotypes in use, most often by the defence to discredit the victim or witness. In some cases, the judge agreed with these stereotypes, while in others, ‘mythbuster’ judicial directions were used. Similarly, Smith and Skinner (2017) also carried out a 10-month observation of 18 rape trials and found that mythbuster directions were also sometimes used but often undermined by the defence in closing arguments, rendering them irrelevant to the jury. This interplay between legal professionals and lay decision making necessitates comprehensive research into the social representations of rape and its current cultural reflections, and how they continue to perpetuate rape stereotypes.

### Demographic Factors

Much research has focused on the demographic predictors of rape myth acceptance, although the greater proportion of the literature centres on gender differences. Such studies have found that men tend to be significantly more likely to accept rape stereotypes than women. This was the case in all countries in a cross-national survey carried out by Fakunmoju et al. (2021) in the United States (US), Nigeria, South Africa, and Ghana.

Additionally, Zidenberg et al. (2021) and Barnett et al. (2018) both carried out studies in which men were more likely to accept rape stereotypes than women, while other factors additionally had an effect, such as religiosity, anti-fatness, and sympathy for victims. It will be useful to gain a similar understanding of gender and rape stereotype acceptance in the UK.

There are fewer studies on other demographics such as ethnicity, sexual orientation, and age. For example, Suarez and Gadalla (2010) carried out a meta-analysis on rape stereotype studies, finding that while ethnic information was often collected, only 6 out of 37 studies contained any comparison between ethnic groups. Studies that have been carried out have found that Black and Asian participants are more likely to accept rape stereotypes than White participants, but generally do not offer explanations as to why this might be. For example, Barn and Powers (2018) found that both Indian and British participants accepted rape stereotypes, but Indian participants showed significantly higher acceptance. However, the authors expressed uncertainty as to what this could be attributed to. Similarly, age as a predictor of rape stereotype acceptance has seen mixed findings. For example, some studies have found that older participants show higher rape stereotype acceptance, such as Adams-Price et al.’s (2004) vignette study, in which the authors attributed the findings to higher levels of conservatism; and Anderson et al.’s (1997) meta-analysis, which also found higher levels of rape stereotype acceptance for older participants. Conversely, Barn and Powers’ (2018) cross-national survey found that younger participants were more likely to accept rape stereotypes, attributing this to an expansion of social networks and life experiences. Therefore, it will be interesting to discover any potential effects of ethnicity and age within the current study.

### Current Study

Due to the detrimental impact that rape and the acceptance of related stereotypes have on both individuals and society, there is a need for systematic research focussing on rape stereotypes and on how rape myth acceptance is impacted by demographic factors. While rape myth acceptance as a topic is not under-researched in general, the authors undertook literature searches which looked for rape stereotype or rape myth studies that were: conducted in England and Wales; representative of the general population; and conducted within the last 10 years, finding 118 articles with ‘general population’ and ‘England and Wales’ specified and 505 articles with only ‘general population’ specified. Not all of these articles were relevant to the search criteria or topic, either in terms of sample size or population, location or topic. Aside from research conducted in Scotland (Prince et al., 2018), there has been no recent, systematic, representative research on rape stereotype acceptance in the UK.

The current study assessed the extent and nature of rape stereotyping in the United Kingdom, using an online anonymous survey. We wished to explore the extent of rape stereotype acceptance in the general population, and also which stereotype categories were adhered to most. The levels of accuracy and uncertainty for the categories as well as individual items were therefore analysed. To gain a deeper understanding of the interplay between demographic factors and rape stereotyping, we carried out inferential analyses between the categories and the demographic information from the survey.

It was first predicted that rape stereotype acceptance would be widespread in the general population, in line with McGee et al., (2002) and McGee et al., (2011) who found in two large-scale telephone survey studies in Ireland that there was a concerning level of agreement with myth statements. It was also predicted that the most accepted categories would be those related to male rape, victims and perpetrators (McGee et al., 2011). Finally, in line with McGee et al. (2011), and also Anderson et al. (1997), whose meta-analysis of 65 articles found that certain demographic variables affected rape stereotype acceptance, it was predicted that men and older people would be most likely to accept rape stereotypes.

## Method

### Participants

An a priori power analysis was carried out in G*Power using a one-way analysis of variance (ANOVA) to determine optimal sample size. Given six groups, which were the six stereotype categories (see below), an effect size of .11, and a power score of .80, the sample was calculated to be 1068. For practical reasons, 1000 was taken as the sample size. 1000 participants were recruited via Qualtrics panelling services. Due to quotas being placed on the groups, the sample was representative of the population of England and Wales in terms of Age, Gender, Ethnicity and Employment Status. Demographic statistics were gathered from Qualtrics’ Census data, which was sourced from Eurostat (2017) (See Table 1 for participant demographics). Participants were reimbursed for their time via Qualtrics with the equivalent of £5 in incentives (such as prizes, sweepstakes and points-based reward programmes).

**Table 1.** Participant Demographics.

	n (%)
Gender	
Male	515 (49.0%)
Female	534 (50.9%)
Non-Binary	1 (0.1%)
Ethnicity	
White British	902 (85.9%)
Asian/Asian British	79 (7.5%)
Black/African/Caribbean British	35 (3.3%)
Mixed/multiple Ethnic Groups	23 (2.2%)
Other Ethnic Groups	11 (1.0%)
Employment Status	
Employed Part Time	158 (15.0%)
Employed Full Time	428 (40.8%)
Self Employed	109 (10.4%)
Unemployed	48 (4.6%)
Student	60 (5.7%
Retired	148 (14.1%)
Looking after Home or Family	48 (4.6%)
Long-Term Sick or Disabled	44 (4.2%)
Other	7 (0.7%)
Sexual Orientation	
Heterosexual	916 (87.2%)
Gay	33 (3.1%)
Lesbian	21 (2.0%)
Bisexual	63 (6.0%)
Pansexual	6 (0.6%)
Other	11 (1.0%)
Education	
None	21 (2.0%)
GCSEs	279 (26.6%)
A Levels	244 (23.2%)
HNC/HND	82 (7.8%)
Bachelor’s Degree	257 (24.5%)
Master’s Degree	116 (11.0%)
PhD	16 (1.5%)
Other	35 (3.3%)
Nationality	
England	997 (95.0%)
Wales	53 (5.0%)

## Materials and Design

An online questionnaire was created with Qualtrics and was distributed via Qualtrics panelling services. The survey created for the questionnaire was partly based on existing research, such as McGee et al. (2002), who created several survey items that are used in the current survey. The same authors categorised their items into five types in a later study (McGee et al., 2011). These categories were used for the current study. More items were generated by gathering information on popular rape stereotypes from rape support websites (e.g., Nottingham Sexual Violence Support Services’ page on rape myths and the ‘myths vs. realities’ page from Rape Crisis England and Wales).

A pilot study was first carried out to test the scale (*n* = 290), with overall results and category results from Cronbach’s Alpha tests indicating generally high reliability with some items removed (α = .89 overall, with most individual categories showing α = .72 or above). Some items were removed or reworded for clarity, or to counter potential response bias, so further reliability tests were carried out on the final version of the scale. After the pilot study, 40 out of 44 items were used in the main study. A split-half reliability test was carried out on all 40 items, resulting in a score of .91, confirming the scale’s high internal reliability.

After thorough research into existing rape myth scales and the pilot study, the final scale constituted six categories and 40 items: Beliefs about Male Rape (e.g., men cannot be raped) which included five items (*α* = .81); Beliefs about Perpetrators of Rape (e.g., most rapes are committed by strangers) which included 10 items (*α =* .82); Beliefs about Consequences of Rape (e.g., date rape is not as traumatic as stranger rape) which included three items (*α =* .35); Beliefs about Rape Victims (e.g., most rape victims are young and attractive) which included nine items (*α =* .91); Beliefs about Motives for Rape (e.g., once a man is sexually aroused, he has to have sex and cannot help himself), encompassing five items (*α =* .71); and Beliefs about Rape Allegations (e.g., allegations of rape are often false), which included 12 items (*α =* .92).

These were compiled into a matrix-style questionnaire via Qualtrics online survey software, using a seven-point Likert scale ranging from 1 = Strongly Agree to 6 = Strongly Disagree, with ‘Don’t Know’ as the 7^th^ point (not included in mean calculations and treated as missing data). Demographic information was collected at the beginning of the survey, including age, gender, sexuality, employment status, education and ethnicity. To determine the extent of rape stereotype acceptance, frequency analyses were carried out to measure levels of accuracy when responding to the items (see Table 2). Participants rated their agreement with each statement on a Likert scale from 1 = strongly agree to 6 = strongly disagree. A 7^th^ point: ‘don’t know’, was included, to measure levels of uncertainty, which were calculated using missing values analyses to study the frequency of ‘don’t know’ answers.

**Table 2.** % Agreement, Disagreement and ‘Don’t Know’ Answers to Statements.

Male rape myths	Agreed (%)	Disagreed (%)	Don’t Know (%)	Correct (%)
Men cannot be raped	5.1	94.2	.7	94.2^35^
Men who are raped must have been acting gay	3.7	94.6	1.7	94.6^38^
Men who rape other men are usually gay	31.5	57.6	11.0	57.6 ^5^
Male victims are generally less emotionally affected by rape than female victims	6.9	89.6	3.6	89.6^27^
Men are physically strong, so can fight off any rape or sexual assault if they really wanted to	10.4	87.9	1.8	87.9^24^
Perpetrator myths				
Most rapes are committed by someone unknown to the victim	10.1	62.3	16.9	62.3 ^7^
Women do not commit rape	9.7	84.1	6.3	84.1^18^
People who were sexually abused as children become abusers themselves	36.3	49.6	14.2	49.6 ^2^
Alcohol, drugs, stress, or depression can turn people into rapists	44.0	42.3	13.6	42.3 ^1^
There is often a ‘type’ of person that commits rape	28.0	59.2	12.8	59.2 ^6^
Most rapes are committed by strangers	19.3	67.0	13.6	67.0 ^8^
Rapists are mostly paedophiles, animals or evil	35.6	55.0	9.3	55.0 ^3^
Rapists are mostly psychotic or mentally ill	34.9	56.8	8.4	56.8 ^4^
Men of certain races and backgrounds are more likely to be rapists	22.4	67.0	10.5	67.0 ^9^
Myths about consequences of rape.				
Date rape is not as traumatic as stranger rape	6.4	89.1	4.4	89.1^26^
It is only rape if someone is physically forced into sex and has the injuries to show for it	11.9	85.4	2.7	85.4^21^
Victim myths				
A woman cannot be raped by her husband	3.8	95.3	.8	95.3^39^
A person could stop a rapist if they really wanted to	8.8	88.3	3.0	88.3^25^
A raped woman is not usually an innocent victim	6.0	90.2	3.7	90.2^29^
Most rape victims are young and attractive	9.5	93.0	5.8	93.0^33^
Women are most likely to be raped after dark by a stranger, so shouldn’t go out at night alone	25.4	69.6	5.1	69.6^11^
Women who are raped often deserve it, especially if they enter a man’s home or car	4.7	94.1	1.2	94.1^34^
Some women have an unconscious desire to be raped	10.0	78.5	11.4	78.5^14^
The victim getting aroused or ejaculating during sexual assault means they probably wanted it	7.2	85.0	7.8	85.0^19^
Prostitutes cannot be raped	4.1	94.5	1.4	94.5^36^
Myths about motives for rape				
Women who wear short skirts/tight tops invite rape	13.4	85.2	1.5	85.2^20^
Rape is only about sex.	12.6	81.4	5.9	81.4^15^
Once a man is sexually aroused, he absolutely has to have sex and cannot help himself	6.1	91.9	1.9	91.9^31^
If a man pays for a dinner or date, a woman should reciprocate with sex	2.7	96.6	.7	96.6^40^
Myths about allegations				
When a woman says no, she is playing hard to get and generally means yes	4.3	94.6	1.0	94.6^37^
If a rape victim isn’t visibly upset by the experience, it probably wasn’t rape	5.4	92.8	1.8	92.8^32^
Accusations of rape are often false	17.7	69.1	13.2	69.1^10^
If the victim drank a lot or took drugs they shouldn’t complain if they ended up being raped or sexually assaulted	8.2	89.9	1.8	89.9^28^
‘Stealthing’ is just a sex trend and is not sexual assault or rape	10.5	75.0	14.5	75.0^12^
‘Real’ victims report rape immediately	12.4	82.7	4.9	82.7^16^
If the case didn’t go to court, the accuser was probably lying	5.8	85.8	4.3	85.8^22^
Abuse in same sex relationships tends to be mutual and both partners’ fault	5.7	83.8	10.5	83.8^17^
Sexual abuse rarely happens in same-sex relationships	5.6	76.2	18.3	76.2^13^
If the person initially consented to sex, but changed their mind and their partner carried on, then it’s not rape	7.1	85.8	5.9	85.8^23^
Transgender people can’t be raped.	2.7	91.5	7.0	91.5^30^

Note: Superscript numerals denote item response accuracy from least accurate at ^1^ to most accurate at ^40^. 11 items were at >75% accuracy, 17 items were at 75-90% accuracy, and 12 items were at >90% accuracy.

The current study uses the term ‘accuracy’, meaning how correct or incorrect the participants are, to align with participants’ rape stereotype acceptance levels. Where participants show lower accuracy when responding to stereotypical statements, for example ‘rape allegations are often false’, this is indicative of higher stereotype acceptance. The reason for this choice was to have an empirical, objective measure of false/true. To measure levels of accuracy, participants who disagreed with false statements and agreed with true statements (reverse coded stereotype statements) were classified as correct. Statements were classified as true or false based on empirical research (McGee et al., 2002) and educational and support websites such as Rape Crisis England and Wales.

### Procedure

The Qualtrics panelling service carried out a ‘soft launch’ of the survey, collecting 10% of the total sample size for review, and then fully launched the survey. Recruitment was carried out in April 2019, and initial data collection took one week, from soft launch to full completion. Participants were sent an anonymous link to the survey, which they clicked to see an information sheet and consent form. After giving full informed consent, they filled in the survey with the option to withdraw at any time. At the end of the survey participants saw a debrief sheet, with contact details for support services, which were also available throughout the survey. The full data was then reviewed for low quality responses. Examples of this include participants intentionally filling out the survey incorrectly by clicking randomly or ‘straightlining’ answers. Responses such as these were replaced. This process took one week.

## Results

### Overall Stereotype Acceptance

Overall, levels of accuracy in statement responses were high, indicating low rape stereotype acceptance. 11 items of the 40 were below an accuracy threshold of 75%, while 17 items were between 75-90% accuracy and 12 items were responded to with over 90% accuracy (see superscript, Table 2). The least accurate item was ‘alcohol, drugs, stress or depression can turn people into rapists’, while the most accurate item was ‘if a man pays for a dinner or date, a woman should reciprocate with sex’. The former item is related to perpetrators, while the latter relates to victims and definitions of rape.

A missing data analysis was carried out to determine levels of uncertainty with the statements (see Table 2). 12 of the 40 items were above 10% uncertainty. The items of lowest uncertainty were ‘men cannot be raped’, and ‘if a man pays for a dinner or date, a woman should reciprocate with sex’, each at .66% uncertainty. The latter item was also the most accurate, suggesting that accurate participants were more certain.

The items of highest uncertainty were ‘Sexual abuse rarely happens in same-sex relationships’ (18.3%), and ‘People who are sexually abused as children become abusers themselves’ (14%). The former item had a high level of accuracy (76.2%), so the uncertainty may be due to the majority heterosexual sample. The latter had one of the lowest levels of accuracy – 49.6% incorrectly agreed with the statement. A pattern is suggested here, as the least accurate item (‘Alcohol, drugs, stress or depression can turn people into rapists’, 42.3%) had the next highest uncertainty (13.6%). This indicates that those items with higher stereotype acceptance appear to also hold the highest uncertainty about the statements.

### Accuracy Within Stereotype Categories

Table 2 indicates that the most widely accepted stereotypes were perpetrator related.

All but two of the 11 least accurate items (<75%) were in this category. One of the remaining items, ‘men who rape other men are usually gay’, is in the male rape category, yet could also be construed by participants as perpetrator related, and so fits the pattern. These findings indicate that stereotypes about perpetrators are more widely accepted than those of other categories. Most items with the highest levels of accuracy fell into the victim or allegation categories, including items from the male rape category that could be included in the victim category. This suggests that participants did not tend to endorse stereotypes about rape victims or the nature of consent.

The perpetrator category held the highest uncertainty, with six out of 10 items over 10%. The categories with the lowest uncertainty were victim stereotypes, with one out of nine items above 10%, and motives for rape, with all items far below 10%. These results support the indication that participants with higher levels of stereotype acceptance are more likely to be uncertain in their beliefs, and also support the conclusion that inaccurate perpetrator-based stereotypes are more widely accepted, while victim-based or allegation-based beliefs are less so.

### Demographic Factors

A Multivariate Analysis of Variance (MANOVA), followed by several Univariate ANOVAs, was carried out to determine whether acceptance of rape stereotypes was significantly affected by demographic group (See Table 3). Dependent variables were the stereotype categories, transformed into their composite scales taken by each item’s median. Independent variables were five demographics: age, education, employment status, sexuality and ethnicity (see Table 3). A separate one-way ANOVA was carried out to determine the effects of gender on stereotype acceptance (see Table 4). Age, education, employment status and sexuality were found to have no significant effect on stereotype acceptance. Several significant effects were found for Gender and Ethnicity.

**Table 3.** Multivariate and Univariate ANOVA statistics for stereotype endorsement by demographics.

Variables	Wilks’ Lambda	*F*	*df*	Error *df*	Sig.	*ηp2*
Age	.96	1.04	24.000	2111.803	.39	.01
Male Rape Myths		2.24	4	1.858	.06	.01
Perpetrator Myths		1.24	4	1.554	.29	.008
Myths about Consequences of Rape		.57	4	.500	.68	.004
Victim Myths		1.66	4	1.053	.15	.01
Myths about Motives for Rape		1.02	4	.758	.39	.007
Allegation Myths		1.88	4	1.376	.11	.01
Education	.91	1.24	42.000	2841.154	.13	.01
Male Rape Myths		2.24	7	1.560	.07	.02
Perpetrator Myths		.80	7	.767	.74	.007
Myths about Consequences of Rape		2.75	7	1.858	.03*	.02
Victim Myths		.45	7	.474	.63	.009
Myths about Motives for Rape		1.31	7	1.448	.05	.02
Allegation Myths		1.02	7	.693	.46	.01
Employment Status	.93	.83	48.000	2980.916	.78	.01
Male Rape Myths		1.29	8	1.073	.24	.01
Perpetrator Myths		2.62	8	3.292	.008**	.03
Myths about Consequences of Rape		.40	8	.348	.92	.005
Victim Myths		1.26	8	.801	.26	.01
Myths about Motives for Rape		.81	8	.602	.58	.01
Allegation Myths		1.30	8	.954	.23	.01
Sexual Orientation	.98	.83	18.000	1711.684	.86	.006
Male Rape Myths		.49	3	.406	.68	.002
Perpetrator Myths		.46	3	.584	.70	.002
Myths about Consequences of Rape		1.48	3	1.291	.21	.007
Victim Myths		.28	3	.179	.83	.001
Myths about Motives for Rape		.32	3	.241	.80	.002
Allegation Myths		.50	3	.366	.68	.002
Ethnicity	.91	2.13	24.000	2111.803	.001**	.02
Male Rape Myths		5.24	4	4.350	.000***	.03
Perpetrator Myths		2.14	4	2.682	.07	.01
Myths about Consequences of Rape		1.42	4	1.239	.22	.009
Victim Myths		5.21	4	3.309	.000***	.03
Myths about Motives for Rape		3.64	4	2.690	.006***	.02
Allegation Myths		1.26	4	.926	.28	.008

*p < .05. **p <.01. ***p < 001.

**Table 4.** One-way ANOVA Statistics for Stereotype Endorsement by Gender.

Variables	*F*	*df*	Error *df*	Sig.	*η2*
Male Rape Myths	3.03	1	2.99	.08	.001
Perpetrator Myths	.48	1	.64	.48	.000
Myths about Consequences of Rape	10.42	1	10.68	.001**	.01
Victim Myths	17.29	1	12.90	.000***	.003
Myths about Motives for Rape	7.56	1	6.47	.006**	.001
Allegation Myths	15.99	1	12.66	.000**	.001

*p < .05. **p <.01. ***p < 001.

#### Gender

A one-way ANOVA with mean plots showed that men were significantly more likely than women to accept stereotypes in the following categories: consequences of rape (F(1, 10.68) = 10.42, p = .001, η2 = .01); victim stereotypes (F(1, 12.90) = 17.29, p < .001, η2 = .003); motives for rape (F(1, 6.47) = 7.56, p = .006, η2 = .001); and allegation stereotypes (F(1, 12.66) = 15.99, p < .001, η2 = .001)

#### Ethnicity

Post-hoc tests showed that Asian British or Black African/Caribbean British participants were significantly likelier to accept rape stereotypes than other ethnicities in three categories: male rape stereotypes (F_(4, 435)_ = 5.24, p < .001, ηp2 = .03); victim stereotypes (F_(4, 3.309)_ = 5.21, p < .001, ηp2 = .03); and motives for rape (F_(4, 2.690)_ = 3.64, p = .006, ηp2 =.02).

These results indicate that some demographic differences in acceptance and endorsement of rape myths exist, most notably in gender and ethnicity.

## Discussion

This uniquely systematic and representative exploration into rape stereotypes across England and Wales’ general population produced several findings that give rise to various recommendations, including shifting social representations and therefore reducing stereotype acceptance within society: and looking at policy and practice within the police and the CPS, specifically concerning jury education. The findings also open up avenues for future research.

### Overall Stereotype Acceptance

In general, accuracy levels were high, with a majority of participants correctly disagreeing with many of the statements, indicating that there was an overall low level of rape stereotype acceptance. This suggests that broadly, social representations of rape are changing in the UK since past studies were conducted here. For example, Prince et al. (2018) discovered high levels of inaccuracy and uncertainty within their sample, indicating higher stereotype acceptance. However, the victimised group in the study was children, potentially making a difference in social representations and stereotype acceptance when compared with adults.

Despite the apparent shift towards attitude change, the levels of inaccuracy and uncertainty are concerning, especially when considering the sample’s age of 18-75, a jury-eligible age group. Overall, 11 items were below 75% accuracy, indicating that within current social representations, erroneous beliefs persist, the most prominent of which was ‘alcohol, drugs, stress or depression can turn people into rapists’. This may be tied to social representations of mental illness, which, despite a more open discourse in recent years, still carries a stigma. Foster (2001) found a general social representation of ‘mental illness’ existed in the press, involving violence, unpredictability and otherness. This has changed little in the last 20 years: Murphy et al. (2013) found negative representations, including links to violence and drugs, still existent within the UK media, while Lloyd (2010) discussed deeply held stigma and stereotypes attached to drug users, linking media representations and public beliefs that drug users are unpredictable and criminal, as with mental illness. There is a common theme here: a social representation that mentally ill people or drug users, are unpredictable, and are therefore likely to rape.

Besides low accuracy, participants also had the second-highest uncertainty for the item linked to mental illness, and many items with the lowest accuracy also had high uncertainty. It is possible that this study captured one moment during a shift in attitudes due to the #MeToo movement gaining wider public attention. Social representations of rape may be changing, meaning we are seeing lower levels of rape stereotype acceptance, and during this potential transitional period, uncertainty while attitudes shift. Szkeres et al.'s (2020) study in the United States found a similar change in attitudes across six months, likely reflecting a change in social representations. However, given the current statistics on prosecution and conviction rates, concern remains about jury acceptance of and uncertainty about perpetrator myths – if jurors do not consider a defendant fitting the ‘real rapist’ stereotype, this could result in erroneous decision making.

### Accuracy Within Stereotype Categories

The perpetrator related category had the lowest accuracy, and therefore the highest acceptance of rape stereotypes. This indicates that social representations of rape perpetrators are still based around that of ‘real rapist’, which involves narratives of otherness and distancing from society. Hindes and Fileborn (2020), in a critical discourse analysis of a high-profile #MeToo case in the Australian press, found the same othering distinction between ‘real’ perpetrator-monster and perpetrators of coercive rape. This narrative exonerates many perpetrators, and discredits and harms the victims, as narrow social representations of rapists often do not fit the suspect. They do not ‘seem’ like a rapist; therefore, the victim must be lying or mistaken. Thus, despite high accuracy for victim blaming or allegation related categories, erroneous social representations of rapists may still be harmful to victims of rape.

Other items in the perpetrator category were of low accuracy, most strikingly the item ‘men of certain races and backgrounds are more likely to be rapists’, which had 22.4% incorrect responses, suggesting that almost a quarter of the sample may still hold harmful and prejudiced beliefs about race and rape. Debauche (2011) suggested that in western society when the rapist belongs to a culturally dominant group, their offence is blamed on their monstrous nature and isolated from the rest of the group. However, when the rapist is part of a minority group within a dominant culture, the rape is blamed on the minority culture. This is seen in the UK media narrative of ‘Muslim grooming gangs’ which influence social representations of both Islam and rape to the detriment of Muslim communities (Cockbain & Tufail, 2020). This consequently influences social representations of rapists to include those of ethnic minorities, deepening xenophobia and causing mistrust, which may be harmful to victims of ethnic minorities in the UK when reporting their rape. Walker et al. (2019) indicates that many UK Black and Minority Ethnic (BME) victims do not report rape, suggesting that the complex links between the social representations of race and rape warrant deeper investigation

### Demographic Factors

Significant differences in stereotype acceptance were found to exist between certain demographic groups, in line with previous findings.

The hypothesis that older people would be significantly more likely to accept rape stereotypes was not supported by the findings. Some previous research suggests that older adults are more likely to accept rape stereotypes than younger adults, which may be due to these groups tending to conservatism – Adams-Price et al. (2004) carried out a study in which three age groups (*n* = 145) read several differing vignettes and measured levels of victim blaming, finding that older respondents were more likely to blame the victims. They suggested this could be attributed to higher levels of conservatism. Anderson et al.’s (1997) meta-analysis found higher levels of rape myth acceptance for older people. However, more recent research has found this relationship to be the inverse. For instance, Barn and Powers (2018) carried out a cross-national survey of 693 participants, finding that younger respondents were significantly likelier to accept rape stereotypes. They attributed this finding to an expansion of social networks and life experiences. This expansion in perspective could assist with a shift in an individual’s social representations. The lack of significant effects in this demographic may be due to this, and indeed, although there was no meaningful significance, mean plots illustrated a tendency for those aged 45+ to accept stereotypes slightly less than those who were younger, potentially lending some small weight to this argument.

Concerning gender differences, men were significantly more likely to endorse stereotypes in consequences of rape, victim related, motive related and allegation stereotypes. This was in line with the hypothesis regarding gender and is in line with existing research. For example, Adolfsson et al. (2018) found in one of several vignette studies that men were more likely to accept rape stereotypes than women, while McGee et al. (2002) found in their large-scale telephone study that men were more likely than women to accept rape stereotypes. This was repeated later by McGee et al. (2011) who found the same effect. Most recently, a study carried out by Zidenberg et al. (2021) exploring the effects of gender and attitudes towards fatness on rape myth acceptance found that men had the highest mean scores for victim blaming, perpetrator sympathy and rape stereotype acceptance. It is highly likely that stereotypical beliefs about and certain social representations of rape are more likely to persist amongst men within patriarchal cultural contexts in which women were historically considered inferior to men. Barnett et al. (2018) carried out a US study in which men were more likely to accept rape stereotypes than women, while religiosity was also significantly and positively correlated with rape stereotype acceptance. Religion and family, in addition to media, are the most common vehicle for perpetuating social representations, and thus rape stereotypes, accounting for this continuing finding.

Asian and Black participants were significantly more likely to accept rape stereotypes than other ethnicities in the categories of male rape stereotypes, victim stereotypes and motives for rape. This is in line with past research. Mori et al. (1995) carried out a survey-based study with Asian and White university students (*n =* 302), finding that Asian participants were more likely to accept rape stereotypes than men, while Varelas and Foley’s (1998) study showed that overall, Black participants were more likely to endorse rape stereotypes than their White counterparts. Barn and Powers (2018), while writing specifically within the context of Indian and British stereotype acceptance, suggested that this could be due to cultural and gender norms, which are perpetuated and upheld through social representations and vary according to cultural context, but expressed that there were other potential attributions such as lack of education that could also be a consequence of these findings. Further research should be undertaken to understand the complex factors at play in this part of the findings, especially in the light of Suarez and Gadalla’s (2010) meta-analysis of rape stereotype acceptance studies, in which they found only six studies out of 73 comparing rape stereotype acceptance across ethnic groups.

### Implications

The results raise various implications for society, policy and practice. A shift in the social representations of rape may be occurring, with less victim blaming and endorsement of ‘real victim’ stereotypes, making clear the value of social representations theory in this field of research, as well as the impact that factors such as time and place have on social representations and stereotypes. This is a positive finding for victim blaming and secondary victimisation. However, there is still a higher level of endorsement of perpetrator related stereotypes, and those related to ‘real rape’. These stereotypes can still be harmful to rape victims. The acceptance of these stereotypes by the media and general population directly influences policy decisions: Aroustamian (2020) carried out a content analysis of media coverage of sexual violence cases, finding that media representations of rape negatively influenced public opinion, while Fox (2013) discussed the various ways that public opinion can affect policymaking, including media coverage, popular blogs and social media. Thus, if the media and public appear to believe certain stereotypes and see little need for policy targeting the related groups, that is, rape perpetrators, then policy will reflect this in turn.

It would be useful to target any educational interventions or campaigns towards the groups most likely to endorse rape stereotypes, including men, and Black or Asian people, although more research, conducted sensitively and with the studied demographics involved in the design and implementation of the study, is needed to understand the factors behind the latter findings. These could come in the form of media campaigns focussing on the most deeply held stereotypes, that is, the ‘real rapist’ or ‘real rape’ stereotypes.

Practice, in both law and policing, is also affected by public opinion. Flowe et al. (2009) argued that media, when used to perpetuate social representations of rape, can influence whether police and jurors believe the victim and consider the perpetrator responsible, while Garza and Franklin (2021) found that male police officers in the US were more likely to accept rape stereotypes than their female counterparts, reflecting the overall finding. Consequently, if perpetrator and real rape stereotypes are accepted within the general population, they may also be accepted amongst the police and legal professionals, additionally affecting jury decision making. This is reflected in recent police and prosecution practice, in addition to the recent statistic that only 1.4% of rapes resulted in a charge or summons (Home Office, 2020), and the fall in convictions by 26% from 2017–18 to 2018–19.

Efforts to solve the problem with prosecution and conviction are underway – for example, the EVAW Coalition’s successful campaign to open an inquest into the low prosecution rate. The resultant reports from this may help the court system, and hence trickle down to the police, although more needs to be known about the nature and extent of stereotype acceptance amongst professionals who deal with rape. It is also vital to change the social representations of rape for potential jurors – the current sample was representative of jurors, and the finding that some demographics were more likely to accept stereotypes, while some stereotypes were more likely to be accepted is a concern. A further initiative to specifically educate juries sitting on rape trials about stereotypes and their impact pre-trial may thus be of help.

### Future Research Directions

There is much potential for further research, as the results have raised questions about the complex interplay between rape stereotyping, social representations, the media, the general public and legal practitioners. It would be useful to carry out further research on the general population’s social representations of rape. For example, a survey of the general population’s social representation of a ‘real rapist’, in addition to considering how to change such representations. A longitudinal study, or a follow-up questionnaire in one-to-two years, to measure levels of rape stereotype acceptance between this study and then would also be helpful to assess changes in social attitudes and representations.

To further investigate the impact of demographic factors, carrying out research into ethnic and cultural differences of rape perceptions to gain an up-to-date understanding of the issue would be useful. Future research should additionally explore a sexually diverse sample’s levels of rape stereotype acceptance, as the current sample was 87.2% heterosexual. This could be useful in targeting education initiatives towards certain groups.

Future research should also encompass the legal system. A survey similar to that of the present research, targeted at professionals who work closely with rape victims and perpetrators, would help gauge stereotype acceptance when compared to the general population. Additionally, investigating UK police stereotype acceptance during interviews with rape complainants to assess stereotyping in this context and the impact this may have on the victim and the case as well as wider societal implications. Results for these studies would help further shape recommendations for legal policymaking and practice.

## Conclusion

The current study highlights that a shift in social representations is taking place, causing rape stereotypes to be accepted less. Some rape stereotypes are still accepted by specific demographics, and social representations, perpetuated through long-standing vehicles such as religion, family and especially media, could be the driving force behind this acceptance. This is especially the case for perpetrator related stereotypes and men. This raises concerns for policy and practice, specifically in terms of juror decision making, and opens up further research directions, such as similar studies in the legal system, and studies within particular demographic groups.
